# Neuromuscular Disorders: Recent Advances and Open Challenges

**DOI:** 10.3390/brainsci16060607

**Published:** 2026-06-02

**Authors:** Vincenzo Di Stefano, Nicasio Rini, Claudia Vinciguerra

**Affiliations:** 1 Department of Biomedicine, Neuroscience and Advanced Diagnostics (BiND), University of Palermo, Via del Vespro 143, 90127 Palermo, Italy; 2 Neurology Unit, Department of Medicine, Surgery and Dentistry “Scuola Medica Salernitana”, University Hospital San Giovanni di Dio e Ruggi d’Aragona, 84131 Salerno, Italy

Neuromuscular disorders (NMDs) encompass a heterogeneous group of acquired and inherited conditions affecting motor neurons, peripheral nerves, neuromuscular junctions, and skeletal muscle [1]. Although progressive muscle weakness remains the most prominent clinical feature, these diseases frequently involve broader multisystemic manifestations, including sensory symptoms, respiratory impairment, fatigue, pain, dysautonomia, cognitive dysfunction, and psychological burden [2]. This complexity makes diagnosis, prognostic stratification, and long-term management particularly challenging, especially in rare diseases requiring dedicated multidisciplinary expertise. In recent years, the field of NMDs has undergone substantial progress [3]. Advances in molecular, immunological, neurophysiological, and imaging techniques have improved diagnostic precision and expanded the identification of disease-specific biomarkers. At the same time, therapeutic innovation has transformed the management of several NMDs [4,5]. This is particularly evident in autoimmune NMDs, such as myasthenia gravis (MG) and immune-mediated neuropathies, where treatment strategies increasingly depend on disease subtype, pathogenic mechanisms, and real-world evidence [6,7]. In inherited muscle diseases, molecular and metabolic advances are also expanding therapeutic perspectives [8]. However, this progress also introduces new challenges. New diagnostic and therapeutic strategies need to be appropriately incorporated into personalized clinical practice. Biomarkers should support diagnosis, prognosis, disease monitoring, and treatment choices [9,10]. Similarly, new therapies should be evaluated for long-term safety, accessibility, costs, treatment sequencing, and real-world effectiveness [11]. This Special Issue aimed to provide an updated overview of recent developments in the diagnostic, prognostic, and therapeutic management of NMDs.

Overview of Published Papers

The contributions included in this Special Issue reflect the broad and multidisciplinary nature of NMDs. Ginanneschi et al. investigated the clinical value of lumbar puncture in amyotrophic lateral sclerosis, showing that routine cerebrospinal fluid abnormalities have limited clinical relevance, whereas neurodegeneration markers, particularly Aβ42, may provide prognostic information on disease progression and survival (Contribution 1). In parallel, Furia et al. reviewed the role of skin biopsy as a minimally invasive approach for assessing cutaneous innervation and pathological protein deposition, highlighting its established value in small-fiber neuropathy and its expanding applications in other neurological disorders (Contribution 2).

Autoimmune neuromuscular disorders represent another central area of this Special Issue. Vinciguerra et al. reported real-world data on mycophenolate mofetil in generalized MG, supporting its potential role as an effective and well-tolerated long-term immunosuppressive option (Contribution 3). Rossini et al. provided a broader immunopathological perspective on generalized MG, emphasizing disease heterogeneity and the increasing relevance of antibody status, thymic pathology, age at onset, and immune mechanisms in guiding treatment strategies (Contribution 4).

The Special Issue also addressed inherited muscle diseases from both experimental and clinical perspectives. Dias et al. explored early taurine administration in the mdx mouse model of Duchenne muscular dystrophy, showing reduced RIP1 levels and suggesting a possible role of necroptosis modulation in dystrophic muscle (Contribution 5). Tizzoni et al. reviewed cognitive, psychological, and psychosocial aspects of Duchenne muscular dystrophy, emphasizing that disease burden extends beyond motor impairment and includes quality of life, adaptation, and family resources (Contribution 6). Finally, Annunziata et al. investigated adherence to non-invasive ventilation in myotonic dystrophy type 1, showing that psychological factors may influence treatment adherence and reinforcing the importance of multidisciplinary care (Contribution 7).

Conclusions and Future Perspectives

The papers collected in this Special Issue reflect the current direction of NMDs. Diagnosis is becoming more accurate, and several biomarkers are now available or under investigation. However, their use in clinical practice should depend on their real ability to improve diagnosis, clarify prognosis, monitor disease activity, or support treatment decisions. Treatment is also changing. In autoimmune NMDs, the choice of therapy is increasingly influenced by the underlying immune mechanism, disease subtype, comorbidities, and previous response to treatment. In inherited and degenerative disorders, advances in molecular medicine are opening new perspectives, but clinical management still depends on disease stage, respiratory involvement, functional status, and long-term follow-up. Future studies should help clarify which biomarkers and treatments are clinically meaningful in daily practice. For this reason, multicenter collaborations, longitudinal cohorts, and real-world data will be particularly important, especially in rare and heterogeneous diseases. At the same time, neuromuscular care cannot be reduced to pharmacological treatment alone. Respiratory support, rehabilitation, psychological aspects, cognitive function, treatment adherence, and quality of life remain essential parts of patient management. In this scenario, the contributions included in this Special Issue highlight the need for a balanced approach, in which diagnostic and therapeutic innovation is combined with comprehensive clinical management.
